# Statistically controlled identification of differentially expressed genes in one-to-one cell line comparisons of the CMAP database for drug repositioning

**DOI:** 10.1186/s12967-017-1302-9

**Published:** 2017-09-29

**Authors:** Jun He, Haidan Yan, Hao Cai, Xiangyu Li, Qingzhou Guan, Weicheng Zheng, Rou Chen, Huaping Liu, Kai Song, Zheng Guo, Xianlong Wang

**Affiliations:** 1 Department of Bioinformatics, Key Laboratory of Ministry of Education for Gastrointestinal Cancer, Fujian Medical University, Fuzhou, 350122 China; 2 Department of Systems Biology, College of Bioinformatics Science and Technology, Harbin Medical University, Harbin, 150086 China; 3 Fujian Key Laboratory of Tumor Microbiology, Fujian Medical University, Fuzhou, 350122 China

**Keywords:** The Connectivity Map, Differentially expressed genes, Drug repositioning, Phenformin, Metformin

## Abstract

**Background:**

The Connectivity Map (CMAP) database, an important public data source for drug repositioning, archives gene expression profiles from cancer cell lines treated with and without bioactive small molecules. However, there are only one or two technical replicates for each cell line under one treatment condition. For such small-scale data, current fold-changes-based methods lack statistical control in identifying differentially expressed genes (DEGs) in treated cells. Especially, one-to-one comparison may result in too many drug-irrelevant DEGs due to random experimental factors. To tackle this problem, CMAP adopts a pattern-matching strategy to build “connection” between disease signatures and gene expression changes associated with drug treatments. However, many drug-irrelevant genes may blur the “connection” if all the genes are used instead of pre-selected DEGs induced by drug treatments.

**Methods:**

We applied OneComp, a customized version of RankComp, to identify DEGs in such small-scale cell line datasets. For a cell line, a list of gene pairs with stable relative expression orderings (REOs) were identified in a large collection of control cell samples measured in different experiments and they formed the background stable REOs. When applying OneComp to a small-scale cell line dataset, the background stable REOs were customized by filtering out the gene pairs with reversal REOs in the control samples of the analyzed dataset.

**Results:**

In simulated data, the consistency scores of overlapping genes between DEGs identified by OneComp and SAM were all higher than 99%, while the consistency score of the DEGs solely identified by OneComp was 96.85% according to the observed expression difference method. The usefulness of OneComp was exemplified in drug repositioning by identifying phenformin and metformin related genes using small-scale cell line datasets which helped to support them as a potential anti-tumor drug for non-small-cell lung carcinoma, while the pattern-matching strategy adopted by CMAP missed the two connections. The implementation of OneComp is available at https://github.com/pathint/reoa.

**Conclusions:**

OneComp performed well in both the simulated and real data. It is useful in drug repositioning studies by helping to find hidden “connections” between drugs and diseases.

**Electronic supplementary material:**

The online version of this article (doi:10.1186/s12967-017-1302-9) contains supplementary material, which is available to authorized users.

## Background

There are many strategies for drug repositioning based on different data such as chemical structural similarities, genetic variation (SNPs-disease correlations, SNPs-drug response) and gene expression profiling [1]. The strategy based on gene expression profiling has the advantage that it does not require a great amount of a priori knowledge on the diseases or drugs [2–4]. An ideal database of gene expression profiles for drug repositioning study should include gene expression profiles of many cell lines representing a diverse range of diseases before and after drug treatments usually for thousands of drugs or candidate drugs. Thus, to create such a data source is a huge project and very costive. As far as we know, currently there are only two such large databases [2]. One is the LINCS database, which, however, has only profiled 978 genes for 25,581 drugs, and the other is the CMAP database which has profiled more than 12,000 genes on mainly three types of cancer cell lines, MCF7, PC3 and HL60, treated with 1309 bioactive small molecules at various concentrations [5]. As an important public data source, the Connectivity Map (CMAP) [3, 6] has been widely applied to study drug repositioning [2, 7, 8] and drug action mechanisms [9, 10]. It has been cited over 940 times in the past 10 years, as shown in the PubMed Central database. However, in the CMAP project, usually only one drug-treated sample was measured for one drug concentration against several control samples. For such small cell line datasets, traditional statistical methods such as the significance analysis of microarrays (SAM) [11, 12] and Student’s *t* test [13] lack power in identifying differentially expressed genes (DEGs) in the treated cells, while the commonly used fold-change (FC) method with an arbitrary cut-off value [14–16] lacks statistical control and tends to get many false discoveries. Therefore, for drug repositioning studies, CMAP adopts a rank-based pattern-matching strategy [3] to build “connections” between disease signature and all gene expression changes caused by drug treatments. However, if all the genes are used, many drug-irrelevant genes participating in building the “connection” may blur the “connection”. Therefore, it is necessary to screen treatment-related DEGs beforehand.

In contrast to the small number of treated samples, there are in total 492, 277 and 229 gene expression profiles of control samples, respectively, for MCF7, PC3 and HL60, which scatter in different experimental batches of CMAP and there are more available in other data sources. Thus, it would be desirable to exploit these valuable control data to aid the differential expression analyses of small-scale treated samples. However, gene expression profiles from different experimental batches or laboratories cannot be directly compared with each other due to experimental batch effects [17, 18]. In addition, different factors such as culturing with drug vehicles, transfecting with control siRNAs or just blank controls may also influence gene expression profiles of a cell line. Recently, we developed an algorithm, RankComp [19], to identify DEGs in an individual cancer tissue through comparing the relative expression orderings (REOs) within a disease sample with the highly stable REOs predetermined in a large collection of normal samples. The algorithm finds up-regulated and down-regulated genes which lead to the disrupted REOs of gene pairs in the disease sample [19].

In this study, by analyzing the gene expression profiles of three types of commonly used cancer cell lines, HepG2, HCT116 and MCF7, we show that the REOs of genes pairs are highly reproducible in the control samples of a particular cell type, even though these samples were collected from different laboratories with different cultivation conditions, but widely disrupted after certain treatments. Based on this observation, we adapted RankComp to datasets with only one technical replicate. The modified algorithm, named OneComp, was evaluated based on data of three cell lines. To demonstrate the usefulness of the OneComp algorithm, we applied it to an application case study on repositioning two drugs, phenformin and metformin, for NSCLC based on the CMAP database.

## Methods

### Data and pre-processing

**Fig. 1:**
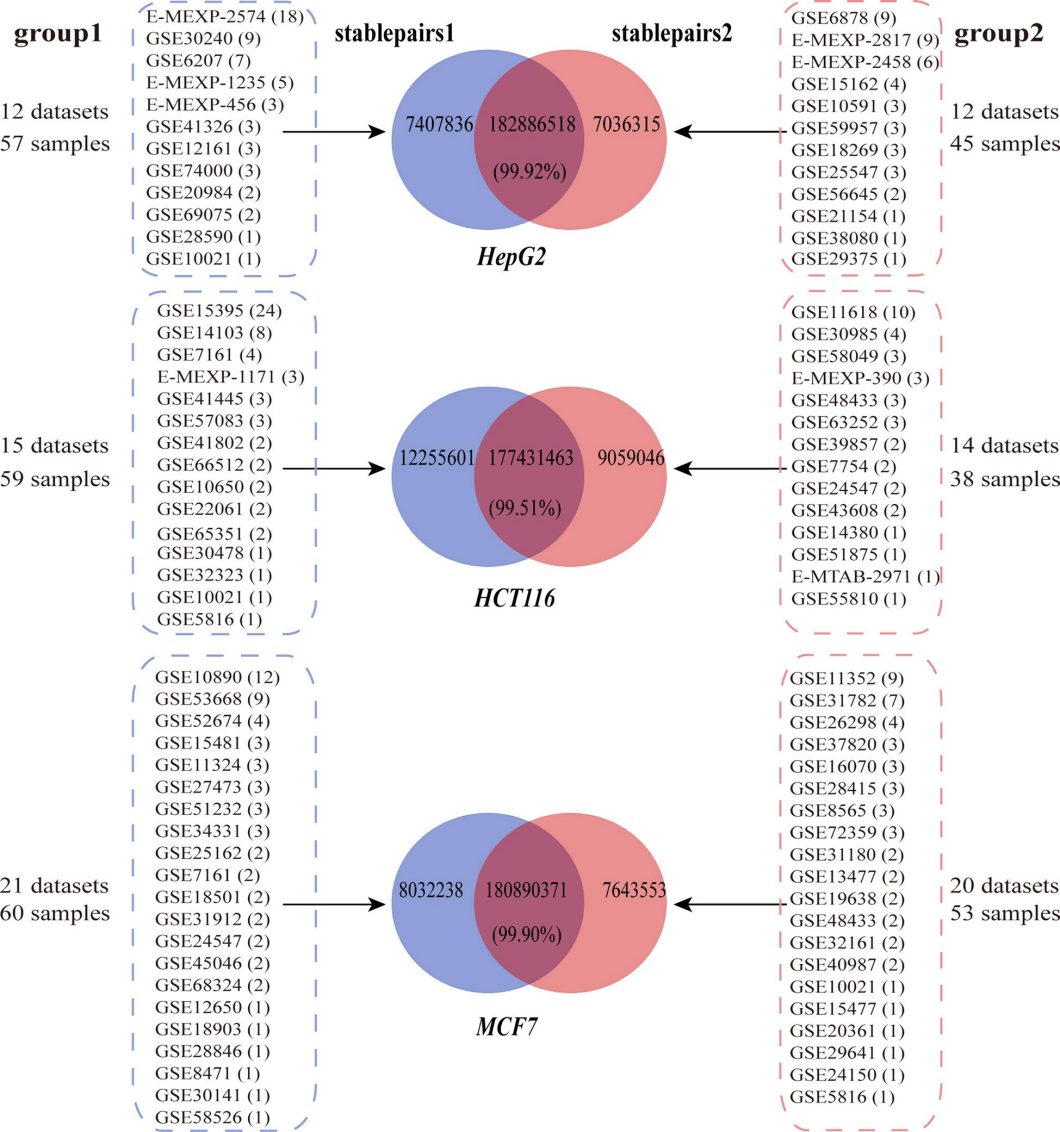
The control samples of HepG2, HCT116 and MCF7 cell line collected from different laboratories. Samples for each type of cell lines were divided into two group, referred to as group1 and group2. Blue pie represents the stable gene pairs1 identified in the group1, red pie represents the stable gene pairs2 identified in the group2. The overlap in the pie represent common gene pairs in the stablepairs1 and stablepairs2 and the number in the brackets represent the consistency score, which denotes the percentage of common gene pairs in stableparis1 and stableparis2 that display the same REO patterns

Control samples of human cell lines HepG2 for liver cancer, HCT116 for colorectal cancer and MCF7 for breast cancer from different laboratories were collected to build a background gene pairs (Fig. 1). Three datasets, GSE41326 [20] for HepG2, GSE7161 [21] for HCT116 and GSE37820 for MCF7 were used to evaluate the performance of OneComp (Table 1). All the above datasets were collected from GEO [22, 23] (http://www.ncbi.nlm.nih.gov/geo/) and ArrayExpress [24] (http://www.ebi.ac.uk/arrayexpress/) databases. For the drug-repositioning study, two non-small-cell lung carcinoma (NSCLC) sample datasets [25, 26] (GSE7670 and GSE10072) were used to build the “query signature” and disease signature for NSCLC (Table 2). A large number of control samples for MCF7, PC3 and HL60 cell lines were collected from CMAP database (Table 2) to build the background stable gene pairs. Datasets of metformin and phenformin treated samples for MCF7, PC3 and HL60 cell lines along their control samples were collected from the CMAP database (Table 3) to build the drug signatures.

**Table 1:** Details of the three datasets used in the OneComp performance evaluation

Dataset	Platform	Phenotype	Sub-datasets	Sample no. (control vs treat)
GSE41326	HepG2	Liver	Sub 1	GSM1014792 vs GSM1014795
			Sub 2	GSM1014793 vs GSM1014796
			Sub 3	GSM1014794 vs GSM1014797
GSE7161	HCT116	Colon	Sub 1	GSM172453 vs GSM172451
			Sub 2	GSM172454 vs GSM172452
			Sub 3	GSM172457 vs GSM172455
			Sub 4	GSM172458 vs GSM172456
GSE37820	MCF7	Breast	Sub 1	GSM928442 vs GSM928445
			Sub 2	GSM928443 vs GSM928446
			Sub 3	GSM928444 vs GSM928447

**Table 2:** Description of the datasets used in the drug repositioning

Source	Platform	Normal/control sample size	Cancer/treated sample size	Tissues/cell type
GSE7670	GPL96	26	26	Lung tissue
GSE10072	GPL96	49	58	Lung tissue
CMAP	GPL96	46	/	MCF7
CMAP	GPL3921	130	/	MCF7
CMAP	GPL3921	125	/	HL60
CMAP	GPL3921	116	/	PC3

**Table 3:** Details of the phenformin and metformin treated cells in the CMAP datasets

Drug	Instance id	Batch id	Concentration (M)	Duration (h)	Cell type	Platform
Phenformin	21	2	0.00001	6	MCF7	HG-U133A
	2350	618	0.0000166	6	HL60	HT_HG-U133A
	2312	642	0.0000166	6	MCF7	HT_HG-U133A
	3622	685	0.0000166	6	MCF7	HT_HG-U133A
	4747	700	0.0000166	6	MCF7	HT_HG-U133A
	3725	681	0.0000166	6	PC3	HT_HG-U133A
	4283	701	0.0000166	6	PC3	HT_HG-U133A
Metformin	1	1	0.00001	6	MCF7	HG-U133A
	2	1	0.00001	6	MCF7	HG-U133A
	3	1	0.0000001	6	MCF7	HG-U133A
	4	1	0.001	6	MCF7	HG-U133A
	61	2a	0.00001	6	MCF7	HG-U133A
	1858	629	0.0000242	6	HL60	HT_HG-U133A
	1694	627	0.0000242	6	MCF7	HT_HG-U133A
	5487	737	0.0000242	6	MCF7	HT_HG-U133A
	1816	628	0.0000242	6	PC3	HT_HG-U133A
	5068	718	0.0000242	6	PC3	HT_HG-U133A

For all the data we used, we downloaded the raw data (.CEL files) and used RMA (Robust Multichip Average) [27] for background adjustment (Bioconductor Affy package). Probe IDs were mapped to Entrenz gene IDs using the corresponding platform files. If a probe set was mapped to multiple or zero genes, the data of this probe set were discarded. If multiple probe sets were mapped to the same gene, the expression value for the gene was summarized as the arithmetic mean of the values of the multiple probe sets.

### Identification of significantly stable REOs in control samples

In a sample, the REO of two genes, *A* and *B*, is denoted as *A* > *B* (or *A* < *B*) if gene *A* has a higher (or lower) expression level than gene *B* (*A* and *B* are used for both gene names and their expression values here). In a large collection of control samples for a cancer cell line, gene pairs with significantly stable REOs are determined by a binomial test [28] as follows,

$$P = 1 - \sum\limits_{i = 0}^{s - 1} {\left( {\begin{array}{*{20}c} k \\ i \\ \end{array} } \right)} (p_{e} )^{i} (1 - p_{e} )^{k - i}$$

where *k* denotes the total number of control samples, *s* denotes the number of samples that have a certain REO pattern (e.g., *A* > *B* or *A* < *B*) in *k* normal samples, and *p*_*e*_ (*p*_*e*_ = 0.5 here) is the probability of observing one of two REO patterns in a normal sample by chance. For the multiple binomial tests, the *P* values are adjusted by the Benjamin and Hochberg method to control the false discovery rate (FDR) [29].

### The consistency score for evaluating the reproducibility of stable REOs

We defined a consistency score to quantify the consistency between two lists of stable gene pairs separately identified from two independent collections of control samples measured by different laboratories for a cancer cell line. For two lists of stable gene pairs, if there are *k* overlapping gene pairs among which *s* pairs show the same REO patterns, the consistency score is the ratio, *s*/*k*. The probability of observing a consistency score of *s*/*k* by chance is evaluated by the binomial distribution model [28].

### The OneComp method

The OneComp method is a customized version of RankComp [19] adapting to one-to-one sample comparison where the original RankComp algorithm fails to identify reliable DEGs, as demonstrated in “Results” section. The major change in OneComp for applications in one-to-one sample comparison is that the selection of gene pairs with stable REOs in the control samples. In RankComp, those gene pairs which have significantly stable REOs in the control samples are selected as the background gene pairs. In OneComp, a list of background gene pairs, which are significantly stable in the collected control samples, are selected and archived for a particular cell line beforehand. Given a pair of control sample and treated sample, each with only one replicate, the prebuilt background gene pairs are screened to exclude those pairs whose REOs are not kept in the current control sample. The remained stable gene pairs are called the customized background gene pairs which are further compared with the treated sample to obtain concordant pairs and reversal pairs.

The following steps are the same as RankComp [19]. Briefly, in a treated cell, the gene pairs which have an opposite REO pattern with the customized background stable gene pairs are defined as the reversal gene pairs. For gene *A*, if its expression level is lower (or higher) than the expression level of gene *B* in the customized background gene pairs but opposite in the treated sample, then this reversal gene pair is supposed to support the up-regulation (or down-regulation) of *A* in the treated sample. Fisher’s exact test is used to test the null hypothesis that the frequency of reversal gene pairs which support the up-regulation of gene A in the treated sample is not different from the frequency of reversal pairs which support the down-regulation of gene A in the treated sample. When the null hypothesis is rejected, gene *A* is judged to be up- or down-regulated in the treated sample compared with the control sample if the frequency of reversal pairs which support the up-regulation of gene A in the treated sample is higher or lower than the frequency of reversal pairs which support the down-regulation of gene A in the treated sample.

The C-language implementation of OneComp and the original RankComp method is available at https://github.com/pathint/reoa [30].

### Performance evaluation of OneComp

The performance of OneComp was evaluated based on three large datasets of HepG2, HCT116 and MCF7 cell lines. For each cell line, we collected a large dataset with several technical replicates of both the control and the drug-treated groups and identified DEGs with SAM. OneComp was applied to identify DEGs from the subsets of the large dataset, each with only a pair of control and treated samples, which were evaluated through comparing with the DEGs identified by SAM in the full large dataset. Those DEGs exclusively identified by OneComp were further evaluated according to the observed genes expression dysregulation directions (up- or down-regulations) between the treated and control samples in the subsets.

The consistency score was also used to quantify the consistency between two lists of DEGs. For two lists of DEGs, if there are *k* overlapping DEGs among which *s* genes have the same dysregulation directions (either up or down), the consistency score is the ratio, *s*/*k*. The probability of observing a consistency score of *s*/*k* by chance is evaluated by the binomial distribution model [28].

### The drug-disease reversal score for drug repurposing

We defined a drug-disease reversal score to reflect the therapeutic effectiveness of a drug to NSCLC based on the hypothesis that the dysregulation directions of the DEGs in the disease signature tend to be reversed after drug treatment if the drug is therapeutically effective to the disease [1]. If *k* is the number of disease signature genes overlapping with the drug signature genes, among which *s* genes could be consistent with drug treatment, then the drug-disease reversal score is defined as (1 − *s/k*). The probability of observing a drug-disease reversal score by chance is evaluated by the binomial distribution model [28].

In CMAP, without the preselection of DEGs induced by a drug treatment, a complex drug-disease scoring scheme is used to evaluate the connection between a drug and a disease, basically based on the same hypothesis as for the reversal score. Readers are advised to see the CMAP website (https://portals.broadinstitute.org/cmap/) for the definition of the score.

### Functional enrichment analysis and protein–protein interaction (PPI) network analysis

We used the GO-function algorithm [31], which is based on the cumulative hypergeometric distribution model, to detect non-redundant GO terms that are significantly enriched for the genes of interest.

Based on the PPI data downloaded from SIGNOR Database [32], the PPI network was built by Cytoscape [33].

## Results

### Stable REOs within a particular type of cell line

As a basis for the applicability of OneComp in the small-scale cell line experiments with only one or two technical replicates, the stability of REOs of gene pairs was evaluated within technical replicates measured by different laboratories for a particular cell line. We collected a total of 102 control samples of HepG2 cell line measured by 24 laboratories which had different cultivation environments (Fig. 1). We randomly divided the datasets from 24 laboratories into two groups each with 12 laboratories’ datasets referred as group1 and group2 (Fig. 1). Then, 190,294,354 and 189,922,833 gene pairs were identified from group1 and group2, respectively, with significantly stable REOs (binomial test, FDR < 1%). Between the two lists of gene pairs, there are 182,886,518 overlapping gene pairs, approximately 96% of the stable gene pairs detected in either group, and more than 99.92% of the overlapping gene pairs also have the same REOs (binomial test, *P* < 1.0 × 10^−16^) (Fig. 1).

Similar results were also observed for the HCT116 and MCF7 cell line samples measured by different laboratories (Fig. 1). These results demonstrate that the significantly stable REOs of gene pairs are highly reproducible across control samples measured by different laboratories for a particular cell type.

### Performance of RankComp and OneComp in cell data with only one technical replicate

**Fig. 2:**
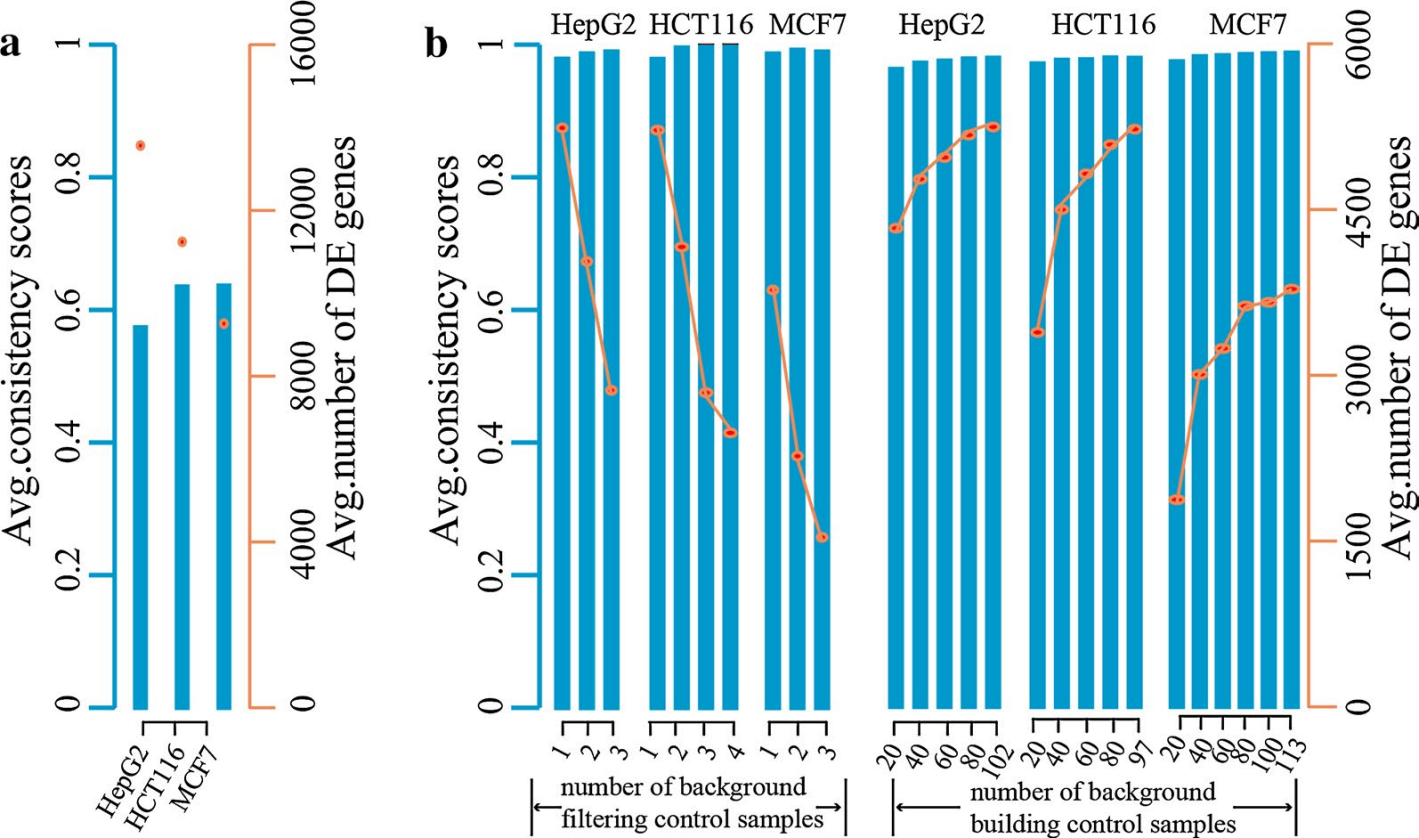
Performance of RankComp and OneComp in cell data. **a** Performance of RankComp in cell data with only one technical replicate. **b** Sample size influence on performance of OneComp via background filtering and building

Based on the above finding, we applied the RankComp to cell data with only one technical replicate. Taking HepG2 cell lines as an example, we identified 194,730,608 gene pairs with significantly stable REOs (binomial test, FDR < 1%) from 102 control samples available in GEO or ArrayExpress, defined as the background gene pairs (Fig. 1). To mimic the data with only one technical replicate, we divided a large dataset (GSE41326) with three pairs of technical replicates for HepG2 transfected with RNF43 siRNA and negative control into three sub-datasets, each with a pair of treated and control sample. Without using the control sample in each of the subsets to filter the background stable gene pairs, RankComp identified 13,566 DEGs on average in the three sub-datasets. However, the average consistency score between the dysregulation directions (up- or down-regulation) of the identified DEGs and the dysregulation directions observed between the treated and control cells is only 57.37%, which indicates a large false positive rate. Similar results were observed for the comparison studies on GSE7161 and GSE37820, the datasets for the HCT116 and MCF7 cell lines, respectively (Fig. 2a). Thus, RankComp is not suitable for datasets with only one sample, indicating that the significantly stable REOs of gene pairs identified across the control samples measured by different laboratories might be still unable to completely exclude the influences of different cultivation environments (Fig. 1).

Therefore, we customized the background stable REOs by filtering out the gene pairs with reversal REOs in the control sample(s) of the analyzed datasets (see “Methods”). This customized version of RankComp is called OneComp. To evaluate the performance of OneComp, we used the DEGs identified by SAM in the above-mentioned three large datasets as a “gold standard”. With FDR < 5%, SAM identified 1896 DEGs in the full GSE41326 dataset while OneComp identified 5247 DEGs on average in the three paired subset samples of GSE41326, which included 56.91% (1079) of the DEGs identified by SAM. Almost all of these overlapping DEGs detected by SAM in the full dataset and by OneComp in the subsets, the consistency scores (see “Methods”) are all higher than 99% (binomial test, *P* < 1.0 × 10^−16^) (Table 4). There are 817 DEGs identified by SAM but not by OneComp, which tend to be those without sufficient expression changes to disrupt REOs. The number of DEGs solely detected by OneComp is much larger, 4168, on average. When the FDR threshold was increased to 20% for the SAM method, 791 of the 4168 DEGs were identified by SAM (Additional file 1: Table S1). This clearly reflects the very low statistical power of SAM when applied to small-scale cell line data sets. The remained DEGs solely identified by OneComp for the three paired samples were further evaluated according to the observed expression difference method. The average consistency score is 96.85% which indicates the dysregulation directions identified by OneComp are mostly correct (Additional file 2: Table S2).

**Table 4:** Overlap and consistency of DEGs detected by OneComp and SAM (FDR < 5%)

Dataset	DEGs by SAM	Sub-datasets	DEGs by OneComp	Overlap	POG (%)	Consistency (%)
GSE41326	1896	Sub 1	5084	1069	56.33	99.91
		Sub 2	5217	1092	57.49	99.82
		Sub 3	5440	1075	56.43	99.53
GSE7161	1280	Sub 1	4770	917	71.64	100.00
		Sub 2	4595	932	72.81	100.00
		Sub 3	4568	890	69.53	100.00
		Sub 4	6973	909	70.94	99.89
GSE37820	633	Sub 1	3734	314	49.76	100.00
		Sub 2	3657	327	51.82	100.00
		Sub 3	3950	321	50.87	100.00

Pair 1, 2, 3, 4 representing paired control and treated technical replicates 1, 2, 3, 4 within each dataset. Overlap denotes the common DEGs detected in each of the pairs by OneComp and the large dataset by SAM. Consistency denotes the percentage of overlapped DEGs that display the same deregulation direction (up- or down-deregulation) between OneComp and SAM (FDR < 5%). P denotes the significance of the consistency (binomial test). POG denotes the percentage of the DEGs identified by SAM (FDR < 5%) that are consistently detected by OneComp ((FDR < 5%)

Similar results were observed for the comparison studies on GSE7161 and GSE37820, the datasets for the HCT116 and MCF7 cell lines, respectively (Table 4, Additional file 1: Table S1, Additional file 2: Table S2). These results suggest that OneComp can be reliably used to detect DEGs in cell line data with only one technical replicate.

### Sample size influence on performance via background filtering and building

If two or more three control samples were used for the background filtering, the number of detected DEGs decreased and the average consistency score remains to be close to 100% with a slight increase (Fig. 2b), which indicates that it would be sufficient to use only one control sample to remove the inconsistent background REOs.

The influence of the sample size in building the background gene pairs was also evaluated on the performance of the algorithm using the above three datasets. From the 102 control samples for HepG2, we randomly extracted subsets of different sample sizes ranging from 20 to 102 with a step size of 20. For each sample size, other than 102 for which all the controls were included, the random sampling experiment and the followed analysis were repeated 100 times. As shown in Fig. 2b, when the control sample size increases from 20 to 102, the average number of detected DEGs increases from 4331 to 5247 gradually, and meanwhile the average precision increases slightly from 96.17 to 97.84%. The same trend was observed for HCT116 and MCF7 (Fig. 2b). These results suggest that the performance of OneComp improves as the background control sample size increases (Fig. 2b). However, when the background control sample size is greater than 40, the performance gain is minor, suggesting a suitable lower bound of control sample size for building the control REOs background.

### Drug repositioning using DEGs detected by OneComp

It was reported recently that antidiabetic biguanide drugs such as phenformin and metformin have therapeutic potential to treat NSCLC [34–37]. Here, we analyzed whether there exists a “connection” between biguanides based on DEGs induced by the drug treatments and NSCLC. As a comparison, the approach recommended by the CMAP project was also used to analyze the “connection” [3, 6].

**Fig. 3:**
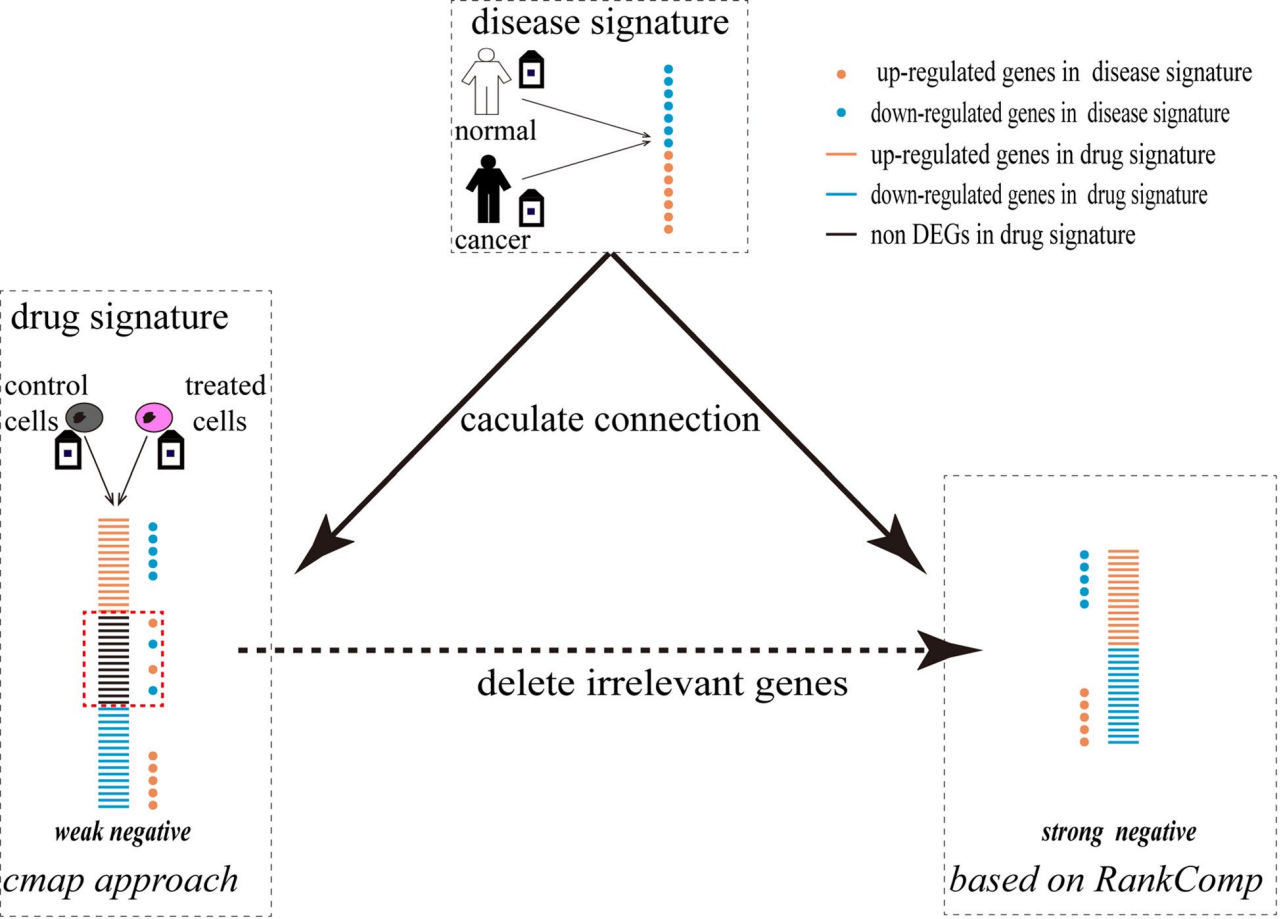
Genes irrelevant to drug treatment may blur the “connection” between a drug and a disease. Blue dots and bars represent the up-regulated genes; red dots and bars represent the down-regulated genes; black bars represent the non-regulated genes

According to the CMAP approach, a “query signature” which includes 10–500 of both up- and down-regulated probe sets should be built first. Using SAM with FDR < 5%, we found 8480 differentially expressed probe sets between NSCLC and lung normal tissues reproducibly in GSE7670 and GSE10072 measured on the HG-U133A platform. Among the differentially expressed probe sets, 641 probe sets were selected with a fold-change (FC) value larger than 2 in both the datasets as the “query signature” of NSCLC. We used this “query signature” to search drugs which have links with NSCLC in the CMAP database through the CMAP portal website (https://portals.broadinstitute.org/cmap/). The results showed that none of the mean drug-disease scores between NSCLC and phenformin were significant (Table 5), providing no support for the therapeutic potential to NSCLC. None of the drug-disease scores between NSCLC and metformin were significant either (Table 5). However, the drug signature used in the CMAP-recommended approach is a list of genes which were obtained according to their fold-changes of gene expressions in the drug-treated sample versus the control sample, which tend to include a large proportion of genes which are irrelevant to drug treatment [16]. This problem may blur the “connection” between the drugs and the disease (Fig. 3).

**Table 5:** Results of the drug repositioning for phenformin and metformin

Methods	CMAP name	Overlap genes	Reversal score	N	*P* value
Approach recommended by CMAP	Phenformin_HL60	/^a^	− 0.7880	1	1
	Phenformin_MCF7	/^a^	0.5070	4	1
	Phenformin_PC3	/^a^	− 0.2880	2	0.9887
	Metformin_HL60	/^a^	− 0.6700	1	1
	Metformin_MCF7	/^a^	− 0.2910	7	0.5039
	Metformin_PC3	/^a^	0.4890	2	1
Approach based on OneComp	Phenformin_HL60	1180	0.4271	1	1
	Phenformin_MCF7	489	0.6094	4	< 0.0001
	Phenformin_PC3	190	0.6053	2	0.0023
	Metformin_HL60	1028	0.5564	1	0.0002
	Metformin_MCF7	1296	0.5872	7	< 0.0001
	Metformin_PC3	122	0.6148	2	0.0071

N present the number of the cell samples treated by the phenformin or phenformin at different dose

^a^CMAP approach do not provide these data

Therefore, we attempted to reduce the influence of the irrelevant genes by focusing on the DEGs induced by drug treatment when assessing the “connection”. Using the HL60, MCF7 and PC3 control samples scattered in different experimental batches of CMAP (Table 2), we identified a list of gene pairs with significant stable REOs (binomial test, FDR < 1%) for each of the three cell lines. If a cell type was measured on different platforms, stable REOs were detected separately for each platform, and the consistent REOs across platforms were used as the final background REOs landscape [38]. With the background landscape, OneComp was used to identify DEGs for each of the phenformin-treated samples with FDR < 5% (Table 5). For each of the three cell types, a common drug signature was defined as the DEGs that have the same dysregulation directions in at least two samples treated by phenformin with different doses. Furthermore, we mapped the aforementioned 8480 probe sets to 5760 genes using the corresponding platform files and considered these genes as the disease signature. The reversal scores were calculated between the disease signature and the drug signatures (see “Methods”). The reversal score between the NSCLC disease signature and phenformin drug signature is 0.6094 for MCF7 cell line and 0.6053 for PC3 cell line, which are significantly higher than expected by random chance (binomial test, *P* < 0.0001 and 0.0023, respectively) (Table 5). Similarly, we observed that all of the three reversal scores between metformin and NSCLC were significant (Table 5). The results indicates the therapeutic potential of both phenformin and metformin to NSCLC, and this conclusion is supported by a few previous studies [34–37].

The NSCLC disease signature in this study was obtained from the lung adenocarcinoma (LUAD) samples since LUAD is the main subtype of NSCLC. To evaluate the influence of disease stages on the drug-disease reversal score, we analyzed two groups of the NSCLC samples according to their therapeutic methods [39, 40], stage II to IIIA mainly treated by surgery combined with chemotherapy, and stage IIIB to IV treated by chemotherapy only. For the six drug-disease reversal scores obtained for the two drugs based on three types of cell lines, five scores support the therapeutic potential of the drugs to the stage II to IIIA patients (*P* < 5%). For the stage IIIB to IV patients, four of the six scores support the therapeutic potential of the drugs (Additional file 3: Table S3). These results suggested the therapeutic potential of phenformin and metformin to NSCLC patients at different stages, although slight differences might exist.

**Fig. 4:**
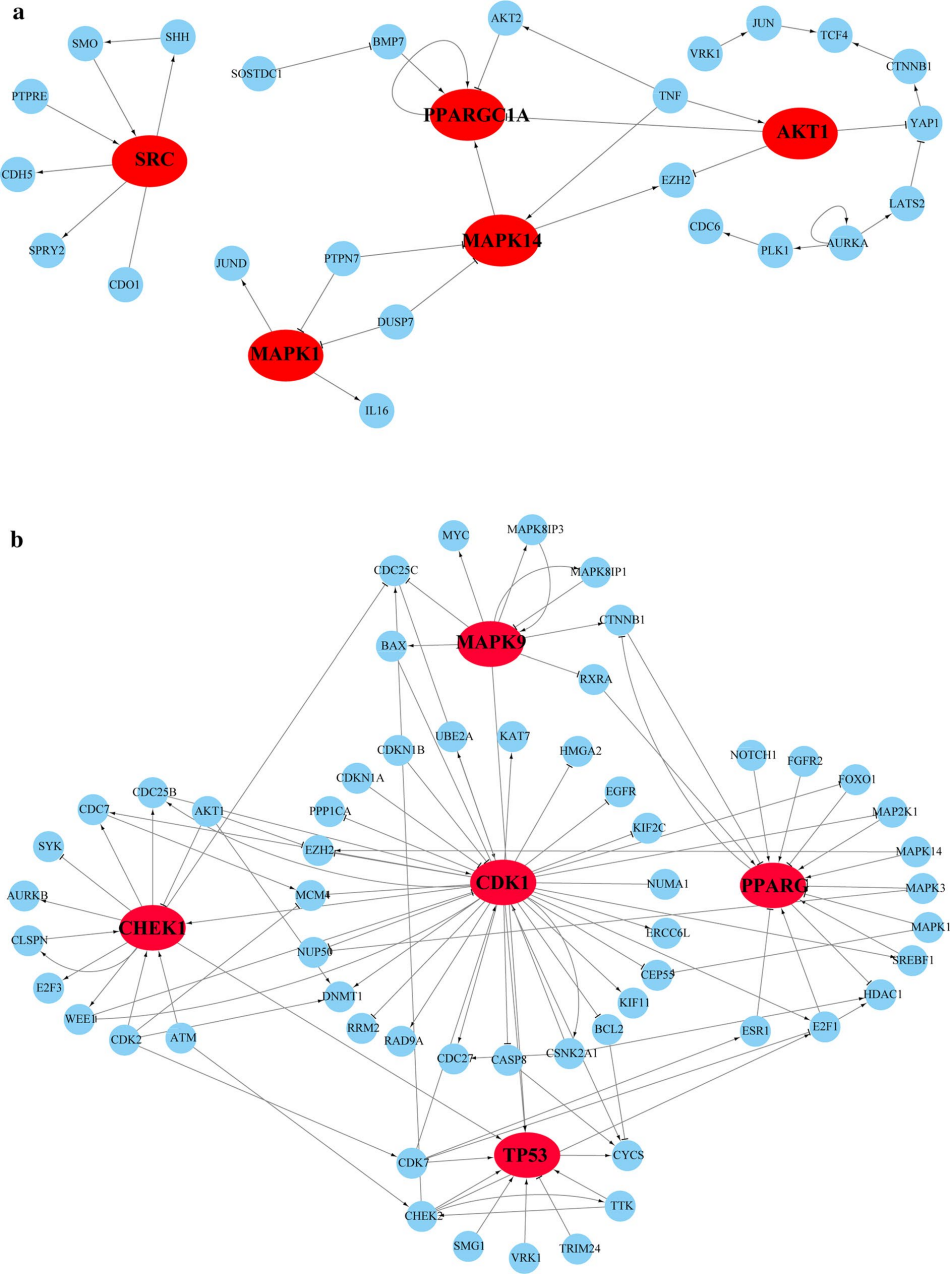
The PPI links between the NSCLC signature DEGs which could be reversed with phenformin treatment (**a**) or metformin treatment (**b**)

If the DEGs up- or down-regulated in the disease signature could be reversed by the drug treatment, they were defined as the drug-target disease genes for the drug. Based on the two list of drug-target disease genes for phenformin and metformin identified from MCF7, which showed the most significant *P* values, we explained the possible anti-NSCLC mechanisms of the two drugs, respectively, through gene ontology (GO) enrichment analysis and protein–protein interaction (PPI) network analysis. The 761 drug-target disease genes for metformin were enriched in six functional categories of GO (hypergeometric distribution model, FDR < 5%), including cell cycle, DNA replication, chromosome condensation and other key cellular processes as described in Additional file 4: Table S4. We further built a one-step protein–protein interaction (PPI) network for these 761 DEGs. As show in Fig. 4a, the top five hub-genes with the highest degrees in the network were MAPK1, MAPK14, PPARGC1, SRC and AKT1 which mainly function in proliferation, cell growth and energy metabolism. The 298 drug-target disease genes associated with phenformin were enriched in no pathway (hypergeometric distribution model, FDR < 5%). This may be due to too few genes. By controlling *P* < 5% instead of FDR < 5%, 16 significant GO functional categories were found which include cell proliferation, cell cycle and other cellular processes as described in Additional file 5: Table S5. Similarly, PPI network analysis showed that the top five hub-genes (MAPK9, PPARG, CHEK1, TP53 and CDK1) are mainly involved in proliferation and cell cycle (Fig. 4b), too. The above results indicate that phenformin and metformin may have therapeutic potential through suppressing the proliferation, growth and energy metabolism of the NSCLC cells [41, 42].

## Discussion

We demonstrated that the REOs of gene pairs are highly stable in control samples measured in different laboratories for a particular cell line. Thus, it is feasible to build a stable REO background landscape for a particular cell line using a large collection of control samples previously measured in different laboratories. For applications to small-scale cell datasets, the stable REO background is customized by filtering out the REOs which are not kept in the control sample(s) of this dataset. Then, OneComp can be used to identify DEGs effectively with statistical control with the customized stable REOs background. Through the analysis on phenformin and metformin treated samples, we showed that the detected DEGs in small-scale cell data with only one or two technical replicates are valuable in the studies for drug repositioning.

Nevertheless, there are also several inherent limitations to use the proposed method for the analysis of small datasets. Although the average consistency scores of the overlapping DEGs between every two technical replicates within GSE41326 are up to 98.20% which indicated that these DEGs with the same dysregulation directions should be true dysregulated genes, there are 42.54% of DEGs on average that were detected in only one technical replicate. The average consistency score of non-common DEGs is only 43.45% if we compare these DEGs identified in one subset with the observed expression difference directions in another subset. This suggests that approximately 32.46% of the overall dysregulated genes detected in one subset of GSE41326 may be unrelated to the biological state of our interest. Similar results were also observed for the GSE7161 and GSE37820 datasets (Additional file 6: Figure S1). Fortunately, based on the assumption that an effective drug should be able to counterbalance the perturbations caused by a disease [3, 6], these unrelated genes will not disturb drug repositioning study deeply in searching the connection between a drug signature and a disease signature. In fact, our method has made much progress compared to the method recommended by the CMAP project as it tends to delete most of the drug unrelated genes (Fig. 3).

In the application case study for drug repositioning, the drug-disease reversal score was used in our method to quantify the connection between a disease signature and a drug signature. An ideal drug repositioning model should use the disease samples in restrictive stages and subtypes to produce the disease signature and use the cell lines corresponding to the disease tissue types to produce the drug signature. However, because the drug treatment profiles in CMAP were performed only on MCF7, PC3 and HL60 cancer cell lines, we used the three drug-signatures separately identified from the drug-treated MCF7 (for breast cancer), PC3 (for prostate cancer) and HL60 (for promyelocytic leukemia cancer) cell lines for each of the two biguanide drugs (phenformin and metformin) to infer the “connection” between the drugs and NSCLC. The underlying hypothesis behind the CMAP project is that there are similar (or significantly overlapped) changes in the gene expression profiles of cell lines obtained from different cancer types exposed to the same drug. Here, we observed that, based on the data for three types of cancer cell lines, two of the three reversal scores between phenformin and NSCLC and all of the three reversal scores between metformin and NSCLC were significant (P < 1%), supporting the therapeutic potential of both metformin and phenformin to NSCLC. This also indicates a high consistency between the drug-signatures from drug-treated MCF7, PC3 and HL60, partially supporting the above-mentioned hypothesis. Nevertheless, this hypothesis needs to be fully addressed by evaluating whether the drug-signatures identified from multiple cell lines for different diseases or the same disease are significantly overlapped if treated by the same drug.

## Conclusions

In this study, we revealed that REOs of genes pairs are highly reproducible in the control samples of a particular cell type. Based on this finding, we customized the RankComp method to the application scenario of identifying DEGs in small-scale cell line experiments with only one or two technical replicates. The method performed well in simulated small-scale cell datasets. Using the DEGs identified by OneComp between antidiabetic biguanide drugs (phenformin and metformin) treated samples and corresponding control samples, we built “connections” between biguanides and NSCLC. The “connections” are statistically significant and support biguanides as potential anti-tumor drugs for NSCLC, while the pattern-matching strategy adopted by CMAP missed these “connections”. Therefore, OneComp method is useful in drug repositioning studies by helping to find more hidden “connections” between drugs and diseases.

## Additional files

**Additional file 1: Table S1.** Overlap of DEGs detected by SAM (FDR < 20%) and OneComp.

**Additional file 2: Table S2.** The consistency of remained DEGs solely identified by OneComp.

**Additional file 3: Table S3.** Drug-disease reversal scores of the drug repositioning for phenformin and metformin for LUAD in different stages.

**Additional file 4: Table S4.** The result of GO gene ontology enrichment of the 761 reversed associated with metformin (FDR < 5%).

**Additional file 5: Table S5.** The result of GO gene ontology enrichment of the 298 reversed associated with phenformin (*P* < 5%).

**Additional file 6: Figure S1.** DEGs overlapping between every two paired technical replicates within GSE41326, GSE37820 and GSE7161. Blue pie represents the DEGs of one paired technical replicates, red pie represents the DEGs of another paired technical replicates. The overlap in the pie represent common DEGs detected in both the two paired technical replicates and the number in the brackets in the overlapping region represent the consistency score, which denotes the percentage of DEGs that display the same dysregulated direction between common DEGs detected in both the two paired technical replicates. The number in the brackets in the blue region represent the consistency scores by using the observed expression differences (up- or down-regulations) between paired treated and control technical replicates in red region as the benchmark to evaluate the DEGs of the blue region paired samples. Similarly, The number in the brackets in the red region represent the consistency scores by using the observed expression differences (up- or down-regulations) between paired treated and control technical replicates in blue region as the benchmark to evaluate the DEGs of the red region paired samples.

## Abbreviations

CMAP: The Connectivity Map
DEGs: differentially expressed genes
REOs: relative expression orderings
NSCLC: non-small-cell lung carcinoma
SAM: significance analysis of microarrays
FDR: false discovery rate
