# Supplementary material for: Statistically controlled identification of differentially expressed genes in one-to-one cell line comparisons of the CMAP database for drug repositioning
Source: J Transl Med. 2017 Sep 29;15:198. doi: 10.1186/s12967-017-1302-9 (PMC5622488; doi:10.1186/s12967-017-1302-9)
Supplement: Supplementary file 1 — Additional file 1: Table S1. Overlap of DEGs detected by SAM (FDR < 20%) and OneComp. [file 12967_2017_1302_MOESM1_ESM.docx]

**Additional file 1: Table S1** Overlap of DEGs detected by SAM (FDR<20%) and OneComp

| Dataset | DEGs by SAM | Sub-datasets | DEGs only by OneComp | Overlap genes | Consistency | P |
| --- | --- | --- | --- | --- | --- | --- |
| GSE41326 | 3786 | Sub 1 | 4015 | 764 | 98.95% | <1.0x10^-16^ |
|  |  | Sub 2 | 4125 | 809 | 98.89% | <1.0x10^-16^ |
|  |  | Sub 3 | 4365 | 801 | 99.13% | <1.0x10^-16^ |
| GSE7161 | 3482 | Sub 1 | 3853 | 1011 | 100.00% | <1.0x10^-16^ |
|  |  | Sub 2 | 3663 | 1010 | 99.80% | <1.0x10^-16^ |
|  |  | Sub 3 | 3678 | 862 | 99.54% | <1.0x10^-16^ |
|  |  | Sub 4 | 6064 | 968 | 98.86% | <1.0x10^-16^ |
| GSE37820 | 1529 | Sub 1 | 3420 | 285 | 100.00% | <1.0x10^-16^ |
|  |  | Sub 2 | 3330 | 329 | 99.70% | <1.0x10^-16^ |
|  |  | Sub 3 | 3629 | 344 | 100.00% | <1.0x10^-16^ |

Here, we overlapped the DEGs identified by OneComp (FDR <5%) but not show in the SAM (FDR<5%) with SAM (FDR<20%).
