# Supplementary material for: Statistically controlled identification of differentially expressed genes in one-to-one cell line comparisons of the CMAP database for drug repositioning
Source: J Transl Med. 2017 Sep 29;15:198. doi: 10.1186/s12967-017-1302-9 (PMC5622488; doi:10.1186/s12967-017-1302-9)
Supplement: Supplementary file 2 — Additional file 2: Table S2. The consistency of remained DEGs solely identified by OneComp. [file 12967_2017_1302_MOESM2_ESM.docx]

**Additional file 2: Table S2** The consistency of remained DEGs solely identified by OneComp

| Dataset | Sample pairs | The remained DEGs | Consistency | P |
| --- | --- | --- | --- | --- |
| GSE41326 | Sub 1 | 3251 | 96.89% | <1.0x10^-16^ |
|  | Sub 2 | 3316 | 96.62% | <1.0x10^-16^ |
|  | Sub 3 | 3564 | 97.05% | <1.0x10^-16^ |
| GSE7161 | Sub 1 | 2842 | 99.05% | <1.0x10^-16^ |
|  | Sub 2 | 2653 | 95.51% | <1.0x10^-16^ |
|  | Sub 3 | 2816 | 95.99% | <1.0x10^-16^ |
|  | Sub 4 | 5096 | 96.35% | <1.0x10^-16^ |
| GSE37820 | Sub 1 | 3135 | 99.01% | <1.0x10^-16^ |
|  | Sub 2 | 3001 | 97.47% | <1.0x10^-16^ |
|  | Sub 3 | 3285 | 98.54% | <1.0x10^-16^ |

The consistency of DEGs identified by OneComp with FDR<5% but not show in the DEGs identified by SAM with FDR<20%.
