# Supplementary material for: Statistically controlled identification of differentially expressed genes in one-to-one cell line comparisons of the CMAP database for drug repositioning
Source: J Transl Med. 2017 Sep 29;15:198. doi: 10.1186/s12967-017-1302-9 (PMC5622488; doi:10.1186/s12967-017-1302-9)
Supplement: Supplementary file 3 — Additional file 3: Table S3. Drug-disease reversal scores of the drug repositioning for phenformin and metformin for LUAD in different stages. [file 12967_2017_1302_MOESM3_ESM.docx]

Additional file 3: Table S3 Drug-disease reversal scores of the drug repositioning for phenformin and metformin for LUAD in different stages

| **Drug and disease connection** | | **Overlap genes** | **Reversal scores** | ***P*-value** |
| --- | --- | --- | --- | --- |
| **Disease stage** | **Drug and cell lines** |  |  |  |
| Stage II to IIIA | Phenformin_HL60 | 884 | 0.4853 | 0.8181 |
|  | Phenformin_MCF7 | 383 | 0.6501 | <0.0001 |
|  | Phenformin_PC3 | 146 | 0.6849 | <0.0001 |
|  | Metformin_HL60 | 822 | 0.5693 | <0.0001 |
|  | Metformin_MCF7 | 1118 | 0.7191 | <0.0001 |
|  | Metformin_PC3 | 92 | 0.6957 | 0.0001 |
| Stage IIIB to IV | Phenformin_HL60 | 582 | 0.4691 | 0.9375 |
|  | Phenformin_MCF7 | 239 | 0.6109 | 0.0004 |
|  | Phenformin_PC3 | 98 | 0.6939 | 0.0001 |
|  | Metformin_HL60 | 533 | 0.6173 | <0.0001 |
|  | Metformin_MCF7 | 667 | 0.5487 | 0.0066 |
|  | Metformin_PC3 | 57 | 0.6140 | 0.0556 |

Here, the disease signatures for different stages LUAD samples obtain from GSE10072, which are DEGs between LUAD samples and normal lung samples identified by SAM (FDR<5%). The drug signatures are same as the drug signatures used in Table5, which are DEGs between drugs treated cells and control cells identified by OneComp (FDR<5%).
