# Supplementary material for: Statistically controlled identification of differentially expressed genes in one-to-one cell line comparisons of the CMAP database for drug repositioning
Source: J Transl Med. 2017 Sep 29;15:198. doi: 10.1186/s12967-017-1302-9 (PMC5622488; doi:10.1186/s12967-017-1302-9)
Supplement: Supplementary file 4 — Additional file 4: Table S4. The result of GO gene ontology enrichment of the 761 reversed associated with metformin (FDR < 5%). [file 12967_2017_1302_MOESM4_ESM.docx]

Additional file 4: Table S4 The result of GO gene ontology enrichment of the 761 reversed associated with metformin (FDR<5%)

| GO ID | Name | FDR |
| --- | --- | --- |
| GO:0002885 | positive regulation of hypersensitivity | 0.0379 |
| GO:0006260 | DNA replication | 0.0001 |
| GO:0007067 | mitotic nuclear division | <0.0001 |
| GO:0051276 | chromosome organization | <0.0001 |
| GO:0051983 | regulation of chromosome segregation | 0.0028 |
| GO:1903047 | mitotic cell cycle process | <0.0001 |
