# Supplementary material for: Statistically controlled identification of differentially expressed genes in one-to-one cell line comparisons of the CMAP database for drug repositioning
Source: J Transl Med. 2017 Sep 29;15:198. doi: 10.1186/s12967-017-1302-9 (PMC5622488; doi:10.1186/s12967-017-1302-9)
Supplement: Supplementary file 5 — Additional file 5: Table S5. The result of GO gene ontology enrichment of the 298 reversed associated with phenformin (P < 5%). [file 12967_2017_1302_MOESM5_ESM.docx]

Additional file 5: Table S5 The result of GO gene ontology enrichment of the 298 reversed associated with phenformin (*P*-value<5%).

| GO ID | Name | *P*-value |
| --- | --- | --- |
| GO:0001568 | blood vessel development | 0.0003 |
| GO:0001570 | vasculogenesis | 0.0010 |
| GO:0003081 | regulation of systemic arterial blood pressure by renin-angiotensin | 0.0019 |
| GO:0006069 | ethanol oxidation | <0.0001 |
| GO:0008283 | cell proliferation | 0.0006 |
| GO:0010518 | positive regulation of phospholipase activity | 0.0007 |
| GO:0030323 | respiratory tube development | 0.0011 |
| GO:0030856 | regulation of epithelial cell differentiation | 0.0002 |
| GO:0032780 | negative regulation of ATPase activity | 0.0014 |
| GO:0042942 | D-serine transport | 0.0006 |
| GO:0044868 | modulation by host of viral molecular function | 0.0017 |
| GO:0048368 | lateral mesoderm development | 0.0014 |
| GO:0060426 | lung vasculature development | 0.0001 |
| GO:0097070 | ductus arteriosus closure | 0.0001 |
| GO:1901671 | positive regulation of superoxide dismutase activity | 0.0017 |
| GO:1902806 | regulation of cell cycle G1/S phase transition | 0.0007 |
