# Supplementary figures and images for: Statistically controlled identification of differentially expressed genes in one-to-one cell line comparisons of the CMAP database for drug repositioning
Source: J Transl Med. 2017 Sep 29;15:198. doi: 10.1186/s12967-017-1302-9 (PMC5622488; doi:10.1186/s12967-017-1302-9)

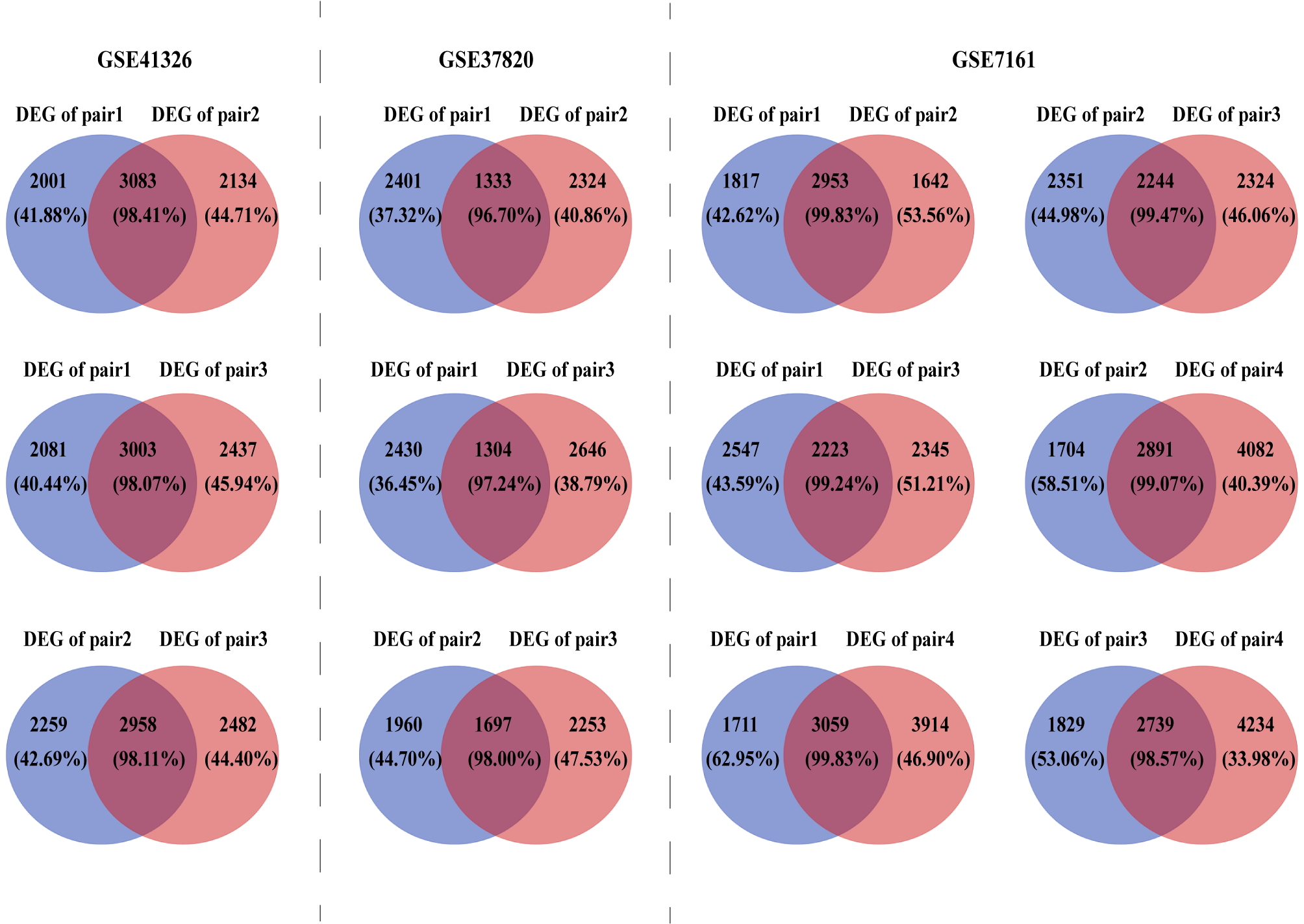

Supplement: Supplementary file 6 — Additional file 6: Figure S1. DEGs overlapping between every two paired technical replicates within GSE41326, GSE37820 and GSE7161. Blue pie represents the DEGs of one paired technical replicates, red pie represents the DEGs of another paired technical replicates. The overlap in the pie represent common DEGs detected in both the two paired technical replicates and the number in the brackets in the overlapping region represent the consistency score, which denotes the percentage of DEGs that display the same dysregulated direction between common DEGs detected in both the two paired technical replicates. The number in the brackets in the blue region represent the consistency scores by using the observed expression differences (up- or down-regulations) between paired treated and control technical replicates in red region as the benchmark to evaluate the DEGs of the blue region paired samples. Similarly, The number in the brackets in the red region represent the consistency scores by using the observed expression differences (up- or down-regulations) between paired treated and control technical replicates in blue region as the benchmark to evaluate the DEGs of the red region paired samples. [file 12967_2017_1302_MOESM6_ESM.tif]
